# Decision-making in the practice of female genital mutilation or cutting in Sudan: a cross-sectional study

**DOI:** 10.1186/s41256-019-0096-0

**Published:** 2019-02-28

**Authors:** Majdi M. Sabahelzain, Ahmed Gamal Eldin, Suad Babiker, Caroline W. Kabiru, Muna Eltayeb

**Affiliations:** 1 Department of Public Health, School of Health Sciences, Ahfad University for Women, P.O. Box 167, Omdurman, Sudan; 2 The Gender and Reproductive Rights Resource and Advocacy Centre (GRACe), Ahfad University for Women, Omdurman, Sudan; 3 Regional Institute of Gender, Diversity, Peace and Rights (RGGDPR), Ahfad University for Women, Omdurman, Sudan; 4 Department of Obstetrics and Gynecology, School of Medicine, Ahfad University for Women, Omdurman, Sudan; 5 Population Council, Nairobi, Kenya; 6 Students Affairs, Ahfad University for Women, Omdurman, Sudan

**Keywords:** Female genital mutilation or cutting, Decision-making, Gender, Sudan

## Abstract

**Background:**

Female genital mutilation or cutting (FGM/C) is a form of violence against women and girls that is widely performed in about 30 countries in Africa, Middle East and Asia. In Sudan, the prevalence of FGM/C among women aged 15–49 years was 87% in 2014. Little is known about household decision-making as it relates to FGM/C. This study aimed to understand the key people involved in FGM/C-related decisions, and to assess predictors of households’ decision to cut or not cut the youngest daughter and the reasons for these decisions.

**Methods:**

We drew on household survey data collected as part of a larger cross-sectional, mixed methods study in Sudan. The analytical sample comprised of data from 403 households that both reported that they had discussion around whether to cut the youngest daughter aged 19 years or younger and arrived at a decision to either cut or leave her uncut. Descriptive statistics summarizing the people involved in FGM/C-related decisions and the reasons for decisions are presented. We also present logistic regression analyses results summarizing predictors of households’ decision to leave the youngest daughter uncut.

**Results:**

Household decision-making on FGM/C involved discussions among the nuclear and extended family, and non-family members. Mothers and fathers were found to be the key decision makers. A greater proportion of fathers were involved in instances where the final decision was to leave the daughter uncut. Thirty-six percent of households decided to leave the youngest daughter uncut. State of residence, mothers’ level of education and FGM/C status and exposure to FGM/C-related information or campaigns were associated with households’ decision to leave the daughter uncut. Health concerns were the most commonly cited reason for deciding not to cut their daughters (57%), while custom or culture was the most commonly cited reason for households deciding to cut their daughter (52%).

**Conclusion:**

FGM/C-related decisions result from deliberations that involve many people. Our findings underscore the important role that fathers play in decision-making and highlight the need to involve men in FGM/C programs. Findings also stress the need to understand and address the drivers of FGM/C.

## Background

Female genital mutilation or cutting (FGM/C) is a form of violence against women and girls that is widely practiced in about 30 countries in Africa, Middle East and Asia [1]. It is estimated that about 200 million girls and women who are alive now have undergone FGM/C, with 3 million girls at risk of being cut each year [2, 3]. FGM/C, which includes all procedures that involve the partial or total removal of the external female genitalia or injury to the female genital organs for nonmedical reasons [4], causes several immediate and long-term health consequences including trauma, excessive bleeding, problems urinating, severe infections, death, and complications in childbirth that may lead to increased risk of perinatal deaths [4, 5].

The prevalence of FGM/C among women aged 15–49 years in Sudan been relatively steady [6]. Between 2006 and 2014, the prevalence of FGM/C among across the country declined slightly from 89 to 87% [7, 8]. The most recent Multi-Indicator Cluster Survey (MICS, 2014) results showed a slight difference between the prevalence in rural and urban areas (87 and 86% respectively) [8]. Prevalence however varied regionally with six states having a prevalence between 94 and 98%, nine states having a prevalence between 78 and 89%, while three states had a prevalence lower than 70%. At 45%, Central Darfur had the lowest prevalence [8].

Most efforts to promote the abandonment of FGM/C in Sudan have been initiated and led by civil society organizations. These efforts have focused on raising people’s awareness about the harmful health consequences of FGM/C, delinking the practice from religion, and calling for legislation against FGM/C [9]. Since 2008, two social marketing campaigns—Saleema and Almawada wa Al Rahma—that aim to change social norms that perpetuate the practice have been adopted and led by governmental bodies including National Council for Child Welfare (NCCW) Ministry of Guidance and Endowment with support from UN agencies [9, 10]. Although there has been little decline in the prevalence of FGM/C among 15–49 year old women [8], there has been progress in terms of changes in attitudes towards FGM/C, the government’s endorsement of the National Strategy for Eradication of FGM/C in a Generation (2008–2018), and the enactment of laws (banning the practice in four states) [9].

Previous studies have shown that the prevalence of FGM/C is associated with various sociodemographic factors. A study that used data from the 2014 MICS in Sudan revealed that low maternal education is associated with a higher likelihood of having undergone FGM/C. Moreover, women from wealthier households were less likely to practice FGM/C than those from poorer households [11]. Studies in Ethiopia have also shown that high mother’s education, urban residence, young maternal age (younger than 40 years) are associated with a lower likelihood of FGM/C, while high socio-economic status, being Muslim and older age are associated with increased odds of FGM/C among women [12, 13].

Although FGM/C in Sudan has been widely studied, there is a paucity of research about the key people who involved in families’ FGM/C-related decisions and the factors associated with families’ decisions to practice or abandon FGM/C. Studies conducted in other countries however reveal the complexities in FGM/C-related decision making. A recent study in Senegal by Shell-Duncan and colleagues revealed the central role played by older women in FGM/C-related decision making and the centrality of social norms, the need to uphold traditions and social hierarchy in households’ decisions to practice or abandon FGM/C [14]. Another study in Sierra Leone showed that FGM/C decision are made mainly by females including grandmothers, mothers and aunts. However, about one-thirds of the participants also noted that fathers were involved [15].

In Sudan, a study conducted in Khartoum in two areas in 2014 showed that many individuals, within the nuclear and extended families are involved in FGM/C-related decisions as are friends, work colleagues, religious figures and local activists, among others [16]. The same study also showed that some family members were more influential than others and that there was a complex web of social, religious, cultural, economic and political factors and experiences that shaped and influenced individual and family views and attitudes towards FGM/C. Families of the same educational, socioeconomic and cultural backgrounds and experiences tended to take different positions on FGM/C. Further, gender power relations, women’s position within their household and the role they play shaped different decisions on supporting or abandoning FGM/C. Previous studies’ findings in Sudan showed that men play very crucial role when the decision about FGM/C is not to cut their daughters [16, 17]. Understanding FGM/C-related decision-making is important for informing the targeting of interventions geared towards abandonment.

## Methods

### Aim, design and study setting

To understand the key people involved in FGM/C-related decisions and assess the predictors of households’ decision to cut the youngest daughter, and the reasons for these decisions, we drew on household survey data collected as part of a larger community-based, cross-sectional, mixed methods study in Khartoum and Gedaref States in Sudan. The large study aimed at investigating the FGM/C decision-making process and the role of gender power relations in Sudan. For further details about the larger study report, see reference [18]. These states were selected because they reflect typical social groups in Sudan and represent a level of diversity in terms of the prevalence of FGM/C, socio-cultural and economic backgrounds and exposure to FGM/C campaign and materials.

In 2014, FGM/C prevalence in Khartoum was estimated at 87.5%, while the prevalence of FGM/C among girls aged 0–14 years was 29.9% [5]. Khartoum does not have an anti-FGM/C law. Khartoum city is the political capital of Sudan where offices of the state, governmental institutions, ministries, embassies and international and regional organizations are located. The prevalence of FGM/C in Gedaref, which borders Ethiopia, was estimated at 78.5% in 2014, while the prevalence of FGM/C among girls aged 0–14 years was 28.9% [5]. Gedaref is home to the largest commercial mechanized farming schemes in Sudan and, consequently, attracts many labor migrants, refugees and internally displaced persons. It is therefore one of the most ethnically, socio-culturally and economically diverse state in the country. While some of these ethnic groups have traditionally practiced FGM/C, for others it is a recent practice stemming from interaction with ethnic groups. While there is currently no federal law that bans FGM/C in Sudan, Gedaref is one of four states where FGM/C is illegal.

Four localities were selected as areas of focus for the study: Jebel Awliya and Umbadda localities in Khartoum State and Gedaref and Al-Faw localities in Gedaref state. These localities were selected as they largely reflect the wide ethnic socio-cultural and economic diversities in the selected state. They have also been targets for FGM/C abandonment programs.

### Study population and sampling

Households were eligible for the survey if they had at least one female aged 7 to 19 years. Households were sampled using a stratified multistage cluster procedure. In the first stage, two localities in each state that are target sites for a large national FGM/C abandonment program were randomly selected. The two localities were then stratified into rural and urban areas. In the next stage, one cluster was selected randomly from each stratum. The number of households to be sampled in each state was determined based on the population density in each state. The households were selected from each cluster using systematic random sampling. Further details about the sampling procedures are provided in the larger study report [18].

Data were collected from 515 households (314 households in Khartoum and 201 in Gedaref States) of which 428 reported that they had discussions around whether to cut the youngest daughter aged 19 years and younger. The analytical sample comprises of data from 403 households that both reported that they had discussion around whether to cut the youngest daughter aged 19 years or younger and arrived at a decision to either cut or leave her uncut. Households that reported a decision to postpone the decision were excluded from the analytical sample (*n* = 25).

### Data collection procedures

In each household, the head of the household was informed about the study and invited to participate in the survey or to nominate someone within the household to answer the survey’s questions. One adult household member was interviewed (about 66% of participants were females) after providing informed consent. In most cases (82% of households), other male and female members of the household were also present during the interview. Data were collected using a pre-tested, structured, paper-based questionnaire. The questionnaire elicited information on the household composition, socio-demographic characteristics of household members, household socioeconomic status, exposure to FGM/C information, attitudes towards FGM/C, household decision-making around FGM/C, and the practice of FGM/C.

### Variables

The dependent variable in this study was the final decision taken by the families (to cut or leave their girl uncut). The independent variables included socio-demographic characteristics of household members which included rural or urban residence, mother’s age, mother’s education’s level, perceived household’s wealth status; and mother’s FGM/C status (cut or uncut). As exposure to FGM/C-related information or campaigns may influence people’s attitudes towards FGM/C [19], we also included exposure to pro-FGM/C information, exposure to anti-FGM/C information, and exposure to any FGM/C-related information as independent variables. Additionally, questions were posed about the key decision makers on FGM/C, and the reasons behind cutting or leaving the girl uncut.

### Management and analysis

Data were double entered using the Statistical Package for Social Sciences (SPSS) software (Version 24) [20]. Bivariate statistics were computed to summarize the descriptive characteristics of the sample and to assess the factors associated with the final decision to circumcise the youngest daughter in the household or not. The decision around FGM/C was measured using a single item referring to the youngest daughter in the household—what was the decision that resulted from the discussion about circumcision? Possible responses were to circumcise the girl, not to circumcise the girl, or to postpone the decision (excluded from the analysis). For the bivariate and multivariate analysis, a binary variable (to cut/not to cut) was created. Variables that were significantly associated with the primary dependent variable at bivariate level were included in a multivariable logistic regression model to identify the predictors of decision-making about FGM/C. A *p*-value of less than 0.05 was considered statistically significant.

## Results

### Characteristics of surveyed households

Table 1 summarizes sociodemographic and FGM/C-related characteristics of surveyed households. It also summarizes the bivariate analysis comparing households based on the final decision around the youngest daughter. Most households (61%) were living in an urban area. Khartoum had a higher proportion of urban households than Gedaref (62 and 37% respectively). Most mothers (58%) were aged between 30 and 49 years. Approximately 44% of the mothers had either primary or *Khalwa* (religious) school. Fifteen percent of mothers had no formal or informal schooling. Majority of the participants (80%) perceived their households as middle-income households. The FGM/C prevalence among the mothers was very high (86%). About one-third of the participants reported exposure to pro-FGM/C campaigns or materials. A greater proportion of participants (74%) reported exposure to anti-FGM/C campaigns or materials. Sixty-four percent (*n* = 256) of households reported that the final decision made was to cut the youngest daughter.

**Table 1:** Households’ characteristics and bivariate associations with FGM/C final decision (*N* = 403)

	Total		Final Decision				
			Cut the girl		Leave girl uncut		*P*-value
	N	%	n	%	n	%	
State							
Gedaref	152	37.7	83	32.4	69	46.9	.004
Khartoum	251	62.3	173	67.6	78	53.1	
Area of residence							
Urban	244	60.5	144	56.3	100	68	.020
Rural	159	39.5	112	43.8	47	32	
Mother’s age							
< 30	71	17.6	41	16	30	20.4	.466
30–39	113	28.0	77	30.1	36	24.5	
40–49	120	29.8	72	28.1	48	32.7	
50 and above	77	19.1	50	19.5	27	18.4	
Missing	22	5.5	16	6.3	6	4.1	
Mother’s education							
None/illiterate	59	14.6	47	18.4	12	8.2	.000
Khalwa (religious school)	31	7.7	19	7.4	12	8.2	
Primary/elementary	148	36.7	103	40.2	45	30.6	
Secondary	91	22.6	46	18.	45	30.6	
University/post graduate	50	12.4	23	9	27	18.4	
Missing	24	6.0	18	7	6	4.1	
Perceived wealth status							
High	16	4.0	8	50	8	50	.013
Medium	323	80.1	197	61	126	39	
Low	62	15.4	49	79	13	21	
Missing	2	0.5	2	100	0	0	
Wealth index							
Very rich	81	20.1	47	18	34	23.1	.323
Rich	65	16.1	37	14	28	19.0	
Middle	95	23.6	63	24	32	21.8	
Poor	81	20.1	51	20	30	20.4	
Very poor	80	19.9	57	22	23	15.6	
Mothers’ FGM/C status							
Cut	347	86.1	228	89	119	81	.023
Not cut	56	13.9	28	10.9	28	19	
Exposure to any pro-FGM/C campaign, information or materials							
No	252	62.5	149	58.2	103	70.1	.018
Yes	151	37.5	107	41.8	44	29.9	
Exposure to any anti-FGM/C campaign, information or materials							
No	103	25.6	84	32.8	19	12.9	.000
Yes	300	74.4	172	67.2	128	87.1	

### Key FGM/C decision-makers in the household

**Fig. 1:**
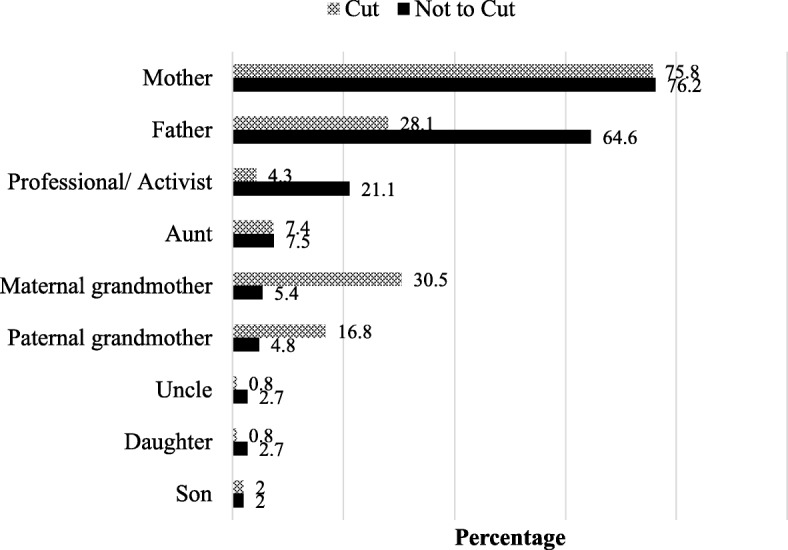
People involved in household’s FGM/C-related decision making

Household decision-making on FGM/C involved discussions among the nuclear and extended family, and non-family members. In almost three-quarters of the households, mothers were reported to be involved in FGM/C-related decisions whether the final decision was to cut the youngest daughter or to leave her uncut (Fig. 1). A greater proportion of fathers were involved in discussions in households where the final decision was to leave the daughter uncut (65%) than in households that decided to cut the daughter (28%). A greater proportion of maternal grandmothers (31%) were involved in decision-making in households that decided to cut the youngest daughter than in households that decided to leave their daughter uncut (5%). About one in five households (21%) that decided to leave their daughter uncut reported that a profession or activist was involved in decision-making.

### Reasons for final decisions to either cut or leave daughter uncut

**Fig. 2:**
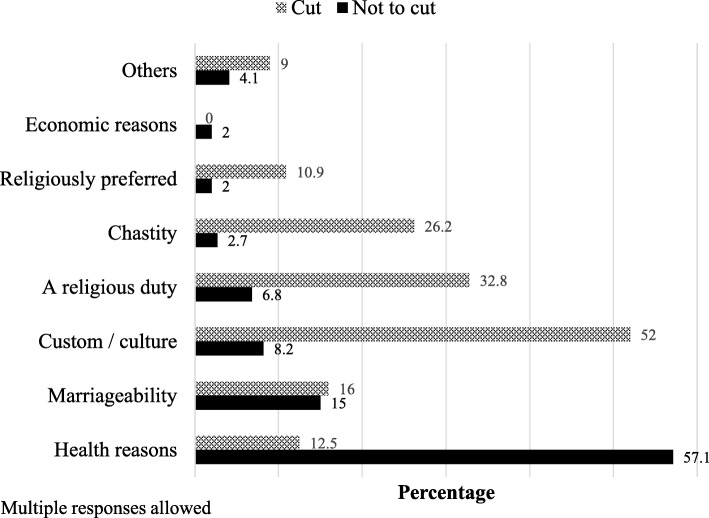
Reasons for final household decision to either cut the youngest daughter or leave her uncut

As shown in (Fig. 2), the majority of household who decided not to cut justified their decision primarily on grounds of health (57%). In contrast, 52% of those who decided to cut their daughter reported that custom or culture was a reason underpinning the final decision. While only 3% of households that decided to leave their daughter uncut reported chastity as a reason for their decision, just over a quarter (26%) of households that decided to cut their youngest daughter did so because of chastity. Religious reasons were also more commonly reported by households that decided to cut their daughter (43.7%) than those who decided to leave their daughter uncut (8.8%). In the larger study [18], qualitative interview respondents often reported that FGM/C is a religious obligation.

### Predictors of the decision not to cut the daughter

To assess the factors associated with the final decision to cut a girl or not, we ran a multivariable logistic regression model including all variables that were significantly associated with the decision to cut or not cut at bivariate level. The logistic regression analysis results are summarized in Table 2. Compared to households with mothers with no education, those with mothers who had been to secondary school and mothers with university and postgraduate education were more likely to decide to leave their daughters uncut (odds ratio (OR) = 3.04 and 3.15 respectively, *p* < 0.05). Households with mothers who had never been circumcised were also more likely to leave their daughters uncut (OR = 2.12, *p* < .05). With regards to the states where the households reside, the households in Khartoum State were less likely as those who lived in Gedaref State to leave their daughter uncut (OR = 0.59, *p* < .05). Rural or urban residence and households’ perceived income were not significant predictors of the final decision. With respect to exposure to FGM/C-related information, participants who reported exposure to pro-FGM/C information were less likely than those who had never been exposed to this information to report a decision to leave their daughter uncut (OR = 0.503, *p* < .05). In contrast, those reported exposure to anti-FGM/C information were approximately two and half times likely to report a decision to leave their daughter uncut (OR = 2.63, *p* < .05). However, exposure to any FGM/C-related materials or information was not a predictor for reporting a decision to leave their daughter uncut.

**Table 2:** Predictors of the decision not to cut youngest daughter

Predictors		95% C.I of OR	
	OR_adj_	Lower	Upper
State			
Gedaref (ref)			
Khartoum	0.588*	.0.351	.0.983
Area of residence			
Urban (ref)			
Rural	0.702	0.420	1.172
Mother’s level of education			
None (ref)			
Khalwa (religious school)	1.821	0.652	5.090
Primary/elementary	1.360	0.625	2.962
Secondary	3.04*	1.356	6.847
University/post graduate	3.15*	1.269	7.824
Perceived wealth status			
High (ref)			
Medium	1.112	0.328	3.768
Low	0.457	0.116	1.805
Mother’s FGM/C status			
Circumcised (ref)			
Not circumcised	2.12*	1.091	4.118
Exposure to any pro-FGM/C campaign, information or materials?			
No (ref)			
Yes	0.503*	0.304	0.831
Exposure to any anti-FGM/C campaign, information or materials?			
No (ref)			
Yes	2.63*	1.200	5.779
Exposure to any anti or pro-FGM campaign, information or materials?			
No (ref)			
Yes	1.07	0.364	3.195

*p < 0.05

*OR*_*adj*_ Adjusted odds ratio, *Ref* Reference category

## Discussion

This study aimed to understand the key people involved in making decisions related to FGM/C in households in two states in Sudan, the predictors of households’ decisions to cut or not cut the youngest daughter in the household, and reasons underpinning the decisions. Although household decision-making on FGM/C involved discussions among the nuclear and extended family, and non-family members, the girl’s parents, particularly mothers, were centrally involved in decisions about whether their daughters would undergo FGM/C. Interestingly, unlike other studies in western African countries that suggest that grandmothers and aunts play a significant role [14, 15], we found that maternal grandmothers were more likely to be involved in households were the final decision was to cut the youngest daughter. Almroth and colleagues [17] in their study in Gezira (Sudan) note that there may be a shift in decision-making of FGM/C with younger parents who question the value of FGM/C independently deciding to leave their daughters uncut.

Further, we found that a significant proportion of fathers were involved in households where the final decision was to leave the daughter uncut. The latter finding mirrors results from studies in Gambia, Sierra Leone and Sudan that found that fathers who were opposed to FGM/C were more likely to be involved in FGM/C-related discussions [15, 17, 21]. The involvement of fathers in decision-making gives credence to recent studies calling for stronger male involvement in FGM/C interventions [22].

Health concerns were the most commonly cited reasons by participants for choosing not to cut their daughters. Given the extensive focus on the health consequences of FGM/C in many abandonment interventions [23], this finding is not surprising. With increases in the medicalization of FGM/C in many countries including Sudan often in response to concerns about the health risks [24], highlighting health concerns in interventions may be a double-edged sword [18, 25]. Program implementers should therefore take measures to raise awareness that medicalized FGM/C does not eliminate the physical, mental and sexual health risks associated with FGM/C.

Our study findings showed that high maternal education was associated with a greater likelihood of households taking a decision not to cut their daughter. These findings suggest that interventions aimed at addressing broader issues like access to education may have an impact on the practice. We also found that households reporting exposure to anti-FGM/C campaigns or materials were more likely to decide to leave their daughters uncut. Previous research in Sudan demonstrates the wide use of multiple channels including mass media and print media to deliver FGM/C abandonment messages [9]. While evidence on the effects of these messages is limited, research conducted in Egypt has found that women who were exposed to FGM/C related messages in printed media, on radio or television, or at community meetings or places of worship were more likely to support the abandonment of the practice [19]. Further research is warranted to understand the characteristics of community members who have limited exposure to FGM/C abandonment campaigns and to identify the most effective ways to deliver FGM/C abandonment messages to these groups.

### Limitations

Study findings should be interpreted in light of the following limitations. Firstly, it was conducted in only two states, which limits generalization to other states. Secondly, the study reports on cross-sectional data and, therefore, causal inferences cannot be made. Thirdly, perhaps because of the time when the interviews were conducted and perceptions that FGM/C is a woman’s issue, majority of participants were female. Women’s views and responses may not reflect the views of men and may introduce unintentional bias. Finally, as noted in the methods section, other household members were present during some of the interviews. The presence of others may have introduced bias in participants’ responses.

## Conclusions

FGM/C-related decisions result from deliberations that involve many people. Our findings underscore the important role that fathers play in decision-making and highlight the need to meaningfully involve men in FGM/C abandonment programs. Findings also stress the need to understand and address possible drivers of FGM/C such as religious norms, which we noted were an important reason for families’ decision to cut their daughters. While increasing awareness of the health risks associated with FGM/C is critical, abandonment programs must also address the medicalization of the practice.

## Abbreviations

FGM/C: Female Genital Mutilation/Cutting
MICS: Multiple Indicator Cluster Survey
OR: Odds Ratio
SPSS: Statistical Package for Social Sciences
